# Vohwinkel syndrome: Clinical and genetic insights from 26 years of follow-up

**DOI:** 10.1016/j.jdcr.2026.04.013

**Published:** 2026-04-27

**Authors:** Yoni Sacknovitz, Celine M. Schreidah, Joshua A. Kent, Azriel Galbut, Aliza Wiederkehr, Ariella Rosenfeld, Todd H. Alter, Larisa J. Geskin

**Affiliations:** a Columbia University Vagelos College of Physicians and Surgeons, New York, New York; b Department of Dermatology, Columbia University Irving Medical Center, New York, New York; c Touro University, Queens, New York; d Center for Dermatology and Skin Surgery, Paramus, New Jersey; e Department of Orthopaedic Surgery, Rutgers Robert Wood Johnson Medical School, New Brunswick, New Jersey

**Keywords:** connexin 26, genetic counseling, germline mosaicism, GJB2, keratoderma hereditarium mutilans, palmoplantar keratoderma, pseudoainhum, sensorineural hearing loss, Vohwinkel syndrome

## Introduction

Vohwinkel syndrome (VS; OMIM #124500), also known as keratoderma hereditarium mutilans, is a rare autosomal dominant ectodermal disorder characterized by honeycomb palmoplantar keratoderma (PPK), stellate keratotic plaques on the dorsal extremities, constricting fibrous bands (pseudoainhum) that may lead to digital autoamputation, and sensorineural hearing loss (SNHL).^1^ Classic VS results from heterozygous mutations in *GJB2*, which encodes the gap junction protein connexin 26, with p.D66H being the most frequently reported mutation.^2^ In the recent pathogenesis-based classification of palmoplantar epidermal differentiation disorders, classic VS is categorized as a GJB2-associated palmoplantar epidermal differentiation disorder (GJB2-pEDD), more specifically GJB2-pEDD-honeycomb keratoderma and pseudoainhum.^3^

While the clinical features of VS are well-characterized, long-term follow-up data remain sparse, particularly in patients who have not received consistent treatment. Most published cases provide cross-sectional descriptions rather than longitudinal follow-up. We report a 26-year follow-up of a patient with genetically confirmed VS who opted against consistent therapy, documenting the cutaneous outcomes, pseudoainhum development and audiologic stability. This case also highlights genetic counseling considerations, including germline mosaicism as an inheritance mechanism and intrafamilial phenotypic variability, that will inform dermatologists managing affected families.

## Case report

**Fig 1:**
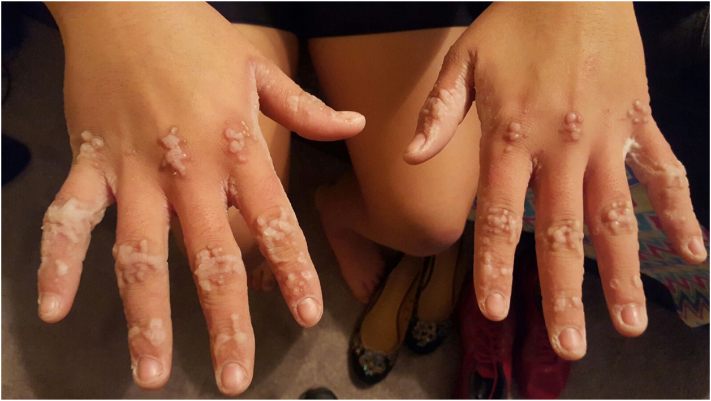
Vohwinkel syndrome. Dorsal hands at age 15, demonstrating stellate keratotic plaques without pseudoainhum of the left fifth digit. Comparison with Figure 4 (age 26) illustrates interval development of a constricting band.

A 26-year-old Hispanic woman presented for evaluation of lifelong palmoplantar keratoderma. She was born at term with diffuse thickening of the palms and soles noted in infancy. These findings were initially misdiagnosed as warts until age 7, when a dermatologist recognized congenital keratoderma. She also failed newborn hearing screening, and audiologic evaluation in early childhood confirmed bilateral high-frequency SNHL. Genetic testing at age 8 identified a heterozygous c.196G>C (p.D66H) mutation in *GJB2*. Notably, targeted sequencing of both parents for the familial mutation was negative, suggesting germline mosaicism in one parent as the most likely inheritance mechanism.^4^ Her younger brother carries the same mutation but per patient report, demonstrates more severe SNHL requiring hearing aids with milder cutaneous involvement. Karyotype and array comparative genomic hybridization were normal. Throughout childhood and early adulthood, she did not receive consistent keratolytic or retinoid therapy, and her palmoplantar keratoderma remained relatively stable (Fig 1). By age 25, she had developed a circumferential constricting band (pseudoainhum) on the left fifth digit at the proximal interphalangeal joint, without ulceration, pain, or vascular compromise.

**Fig 2:**
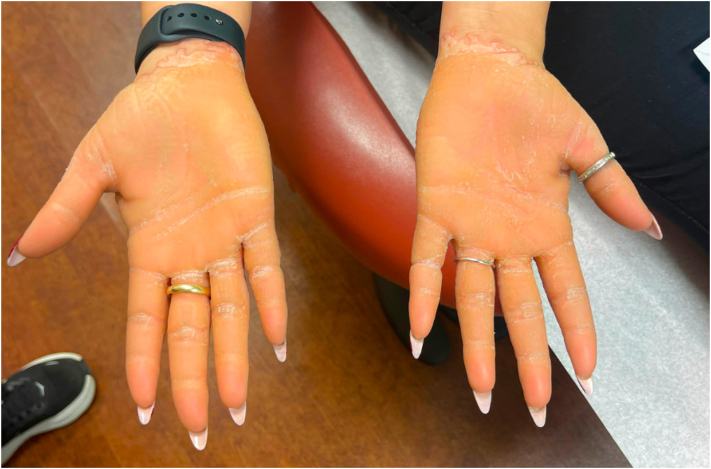
Vohwinkel syndrome. Palmar hyperkeratosis demonstrating honeycomb pattern after 26 years without consistent keratolytic therapy.

**Fig 3:**
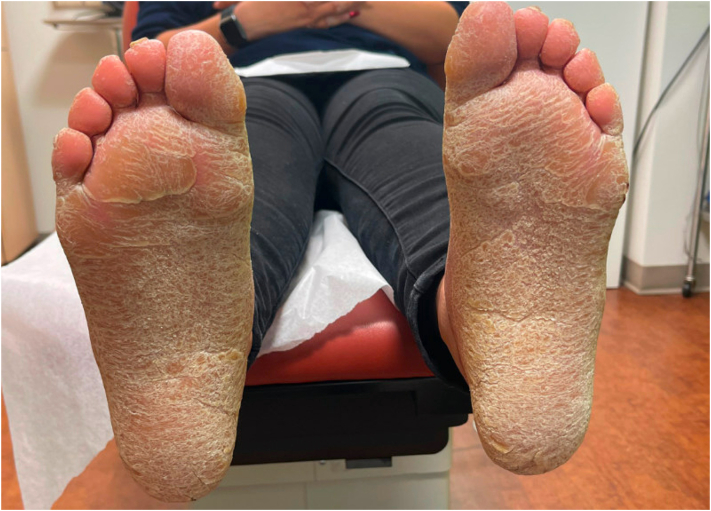
Vohwinkel syndrome. Plantar keratoderma with fissuring on weight-bearing surfaces.

**Fig 4:**
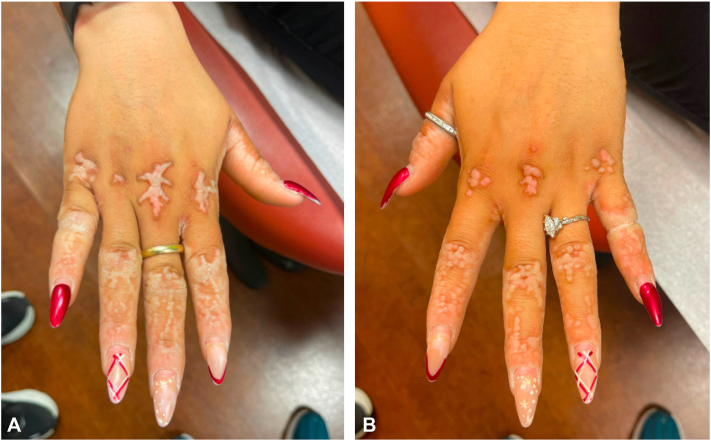
Vohwinkel syndrome. **A,** Stellate (starfish-shaped) keratotic plaques on the dorsal hands, a characteristic feature of the syndrome. **B,** Pseudoainhum (circumferential constricting band) on the left fifth digit at the proximal interphalangeal joint.

On examination, physical findings included bilateral palmar and plantar hyperkeratosis with a honeycomb pattern (Fig 2 and 3). Stellate keratotic plaques were present on the dorsal hands (Fig 4). A circumferential constricting band (pseudoainhum) had developed on the left fifth digit at the proximal interphalangeal joint (Fig 4), without ulceration, pain, or vascular compromise. She had no clinically evident alopecia, nail dystrophy, or other extra-palmoplantar ectodermal abnormalities. The patient was evaluated by hand surgery, and surgical intervention was recommended given impending neurovascular compromise, contraindication to retinoid therapy due to family planning.^5^

Serial audiometry spanning 23 years documents the evolution of her hearing loss. At age 2, auditory brainstem response under sedation revealed mild-to-moderate loss at 2000-4000 Hz with absent otoacoustic emissions. By age 7, pure tone audiometry demonstrated normal thresholds through 1000 Hz with severe sloping loss above 2000 Hz. Most recent testing at age 25 shows stable mild-to-moderately-severe sloping SNHL bilaterally, with speech recognition thresholds of 35 dB and word recognition scores of 90% (right) and 80% (left) at 75 dB HL. Despite documented high-frequency hearing loss, she reports functional hearing in daily activities.

## Discussion

This case offers several insights relevant to dermatologists managing VS, drawing on 26 years of follow-up in a patient who opted against consistent therapy.

First, the development of pseudoainhum despite relatively stable PPK underscores the need for digit surveillance in all VS patients. Despite opting against keratolytic or retinoid therapy, our patient's PPK. remained stable without significant functional impairment. However, pseudoainhum developed by age 26, demonstrating that constricting fibrous bands may progress independently of PPK. No standardized surveillance protocol exists for VS-associated pseudoainhum; our case supports annual digit examination with attention to early band formation, particularly at the fifth digits which are most commonly affected.^6^

Second, the serial audiometric data add to our understanding of hearing loss trajectory in VS. Published descriptions characterize the SNHL as “mildly progressive,” but our patient's high-frequency loss has remained stable over 23 years of documented follow-up.^7^ Her preserved speech discrimination (80% to 90%) allows functional hearing without amplification. This longitudinal data, rarely available in published VS cases, may provide additional information when counseling newly diagnosed families about audiologic prognosis.

Third, this case illustrates 2 distinct genetic counseling considerations. The confirmed germline mosaicism in 1 parent has implications for recurrence counseling: both parents tested negative for the p.D66H mutation on peripheral blood testing, yet 2 children are affected. This suggests that the mutation arose in the parental germline, conferring up to 50% recurrence risk for any future pregnancies despite negative parental blood-based testing.^8^ Dermatologists who diagnose VS should consider referral to medical genetics for appropriate genetic and reproductive counseling. Separately, for affected individuals like our patient who carry a confirmed *GJB2* mutation, standard autosomal dominant inheritance applies, with her future children facing a 50% risk of inheriting the mutation.

Finally, the intrafamilial phenotypic variability reported between siblings—our patient with predominant cutaneous findings versus her brother with predominant SNHL—illustrates the variable expressivity characteristic of *GJB2* mutations.^9^^,^^10^ Identical mutations can produce divergent phenotypes even within families, likely reflecting modifier genes or epigenetic factors that remain poorly understood.

In conclusion, this 26-year follow-up of VS in a patient who opted against consistent therapy demonstrates that while cutaneous disease may not progress without aggressive intervention, pseudoainhum can still develop and requires surveillance. The audiologic stability observed provides valuable prognostic information for patient counseling. Dermatologists should be aware of germline mosaicism as an inheritance mechanism and its implications for genetic counseling in affected families.

## Conflicts of interest

Geskin has served as an investigator for J&J, Mallinckrodt, Kyowa Kirin, Soligenix, Innate, Incyte, Trillium, Merck, BMS, and Stratpharma and on the scientific advisory board for SciTech and Citius.
